# A simple high‐throughput protocol for the extraction and quantification of inorganic phosphate in rice leaves

**DOI:** 10.1002/aps3.11395

**Published:** 2020-10-30

**Authors:** Sompop Pinit, Supachitra Chadchawan, Juthamas Chaiwanon

**Affiliations:** ^1^ Center of Excellence in Environment and Plant Physiology Department of Botany Faculty of Science Chulalongkorn University Bangkok 10330 Thailand; ^2^ Program in Biotechnology Faculty of Science Chulalongkorn University Bangkok 10330 Thailand

**Keywords:** inorganic phosphate, microplate, molybdenum blue assay, rice

## Abstract

**Premise:**

Phosphorus (P) is an essential macronutrient that is often limited in agricultural systems. Determining inorganic phosphate (Pi) contents of plant tissues is crucial for evaluating plant P status. Here, we present a simple, high‐throughput colorimetric microplate technique to measure Pi contents in rice (*Oryza sativa*) leaf tissues, based on the molybdenum blue reaction.

**Methods and Results:**

We used a hole puncher to sample small equal areas of leaf tissue for Pi extraction. We removed the leaf grinding and weighing steps, which are time‐consuming and normally required to release Pi from the tissues and to measure the biomass for data normalization, respectively. We showed that the punching method yielded comparable results to the conventional grinding method for two rice cultivars grown under various levels of P supply.

**Conclusions:**

Compared with existing techniques, this protocol is more suited to an initial screening, enabling one researcher to determine the Pi contents of thousands of rice leaf samples within a few hours.

Phosphorus (P) is an essential macronutrient for plants, and is often the limiting factor in agricultural production. In soils, P is typically fixed with other minerals, making it unavailable for plant uptake. Phosphorus deficiency has been reported to reduce biomass; however, it increases the root‐to‐shoot ratio and alters the root system architecture, enabling plants to explore the soil and increase their nutrient uptake efficiency (Dogbe et al., 2015; Nishigaki et al., 2019). Despite its impact, plants may not show obvious visual signs of P deficiency in their leaves unless they have experienced a severe deficiency for an extended period of time (Frydenvang et al., 2015). Measuring the inorganic P (Pi) contents in the leaves is thus a direct and sensitive method of determining plant P status, which is important for determining optimal P fertilizer applications, as well as for screening plant varieties for high P use efficiency.

The concentration of Pi in a solution is commonly determined using the molybdenum blue reaction, in which orthophosphate (PO_4_^−3^) and molybdate are reacted and reduced to form the phosphomolybdenum blue complex, which is detected spectrophotometrically (Nagul et al., 2015). Such colorimetric techniques can be automated to handle a large number of samples in a high‐throughput manner; however, the current methods of Pi extraction from plant samples typically involve several tedious steps, including grinding, weighing samples, and centrifuging, which render the entire Pi determination process relatively low‐throughput (Mori and Nakamura, 1959; Turner and Turner, 1961; Nanamori et al., 2004; Zhu et al., 2018). To obtain an accurate determination of Pi content, it is critical to know the amount of plant tissue used for the extraction. In general, the sample biomass is quantified and used for data normalization (Kanno et al., 2016), but determining sample fresh or dry weights is time‐consuming, and water loss from fresh tissue during harvesting and weighing could affect the accuracy of the analysis. Furthermore, existing plant Pi extraction protocols generally include a grinding step, which could also be time‐consuming if a multiple‐sample homogenizer is not available.

In this study, we present a simple protocol of Pi extraction and quantification. We used a hole puncher to harvest small equally sized disks of leaf tissue directly into 96‐well plates, removing the need for the grinding and weighing steps used previously. By performing the Pi extraction and the molybdenum blue reaction in 96‐well plates and the measurement using a microplate reader, one researcher could quantify the Pi contents of ~2000 samples in 3 h (hands‐on time, excluding one 3‐h incubation step). We evaluated the accuracy of this method by analyzing two varieties of rice (*Oryza sativa* L.) with different Pi accumulation abilities grown under different P concentrations. We compared these results with those determined using a conventional grinding method (Nanamori et al., 2004). Our results showed that the proposed punching method can be applied to determine a wide range of Pi contents in leaf samples and yielded results comparable to the conventional grinding method.

## METHODS AND RESULTS

### Plant materials and growth conditions

Based on our initial screen of leaf Pi contents in 219 Thai rice cultivars (*Oryza sativa* subsp. *indica* S. Kato) (unpublished results), we selected two, Leuang Chumpae and Nah Khwan, that showed high and low levels of Pi accumulation, respectively, for use in the evaluation of the method presented here. The seeds were sterilized using commercial bleach (2% sodium hypochlorite), rinsed three times with distilled water, and then soaked in distilled water for two days, followed by a pre‐cultivation in half‐strength Yoshida solution (Yoshida et al., 1976) for six days. The seed endosperm was removed from the seedlings to prevent it from contributing any nutrients to the developing plant. The seedlings were then subjected to various P treatments by transferring them into full‐strength Yoshida solution containing different P concentrations for 16 days. The P treatments were 320, 160, 80, 16, and 0.8 µM NaH_2_PO_4_, with the reductions in NaH_2_PO_4_ being compensated with an equal concentration of NaCl. The culture solution was renewed every four days, and the pH was adjusted to 5.8 daily. The experiments were performed in a greenhouse (30–38°C day/26–30°C night; 40–70% day humidity/70–90% night humidity; 11 h of natural light [400–1900 µmol m^−2^ s^−1^] per day) using a completely randomized design. The Pi contents were evaluated using both the punching and grinding methods.

The statistical analysis was performed using an analysis of variance (ANOVA), and the mean comparison was performed with Duncan’s multiple range test in SPSS version 22 (IBM, Armonk, New York, USA). A linear regression was performed using R software (version 3.6.1; R Core Team, 2019).

### High‐throughput punching method

Equally sized leaf samples (leaf disks) were taken using a hole puncher with a 3‐mm diameter (7.07 mm^2^), a size which was selected because rice leaves at the seedling stage are quite narrow. To expedite the punching and harvesting, we folded a leaf in half twice, placed it over glossy paper, and punched the leaf and the paper together. The glossy paper helped to support the soft leaf and prevented the leaf disks from sticking in the puncher hole. It was thus possible to make up to four leaf disks with a single punch.

The leaf disks of each sample were then immediately transferred into individual wells of a 96‐well plate, which was stored in a container filled with dry ice to avoid the hydrolysis of organic P. The plates and samples were covered with aluminum foil and stored at −80°C for the further experimental steps (Appendices 1, 2). In addition, storing leaf disks in below‐freezing temperatures is a freeze‐shattering technique known to permeabilize plant cell walls, thus eliminating the need to grind them to extract Pi from the cells (Wasteneys et al., 1997).

To extract Pi, 200 µL of 5.5% (w/v) perchloric acid was added to each well of a 96‐well plate containing the leaf disk samples, which was then incubated on ice for 3 h. The leaf disks were submerged in the solution during the incubation. The supernatants were then transferred to a new 96‐well plate using a multichannel pipette and diluted with 5.5% (w/v) perchloric acid to a final volume of 80 µL. The diluted supernatants were then used for the Pi measurement.

### Conventional grinding method

The conventional Pi extraction and measurement technique used was as described by Nanamori et al. (2004), with slight modification. A 50‐mg sample of frozen leaf was ground in 100 µL of 10% (w/v) perchloric acid, after which the homogenate was diluted 10 times with 5% (w/v) perchloric acid, incubated on ice for 30 min, and then centrifuged at 10,000 × g for 10 min at 4°C. The supernatant was used for the Pi measurement.

### Measurement of Pi concentrations using the molybdate blue reaction

Molybdate blue reagent, containing 0.4% (w/v) ammonium molybdate in 0.5 M H_2_SO_4_ (solution A) with 10% ascorbic acid (solution B) (A : B = 6 : 1), was added to the supernatant (supernatant : molybdate blue reagent = 1 : 2) and incubated at 40°C for 20 min. The absorbance was measured at a 820‐nm wavelength using a microplate reader. The Pi content was analyzed by comparing the absorbance with the standard curve, and the Pi concentration was calculated in nanomoles per leaf disk area (square millimeters) or micromoles per gram fresh weight.

The Pi concentrations used to generate a standard curve should be in the range of 0.1–40 µg/mL (equivalent to 0.74–294.12 µM). As the Pi contents in the extracts of P‐sufficient samples (320 μM P treatment) could be 100‐fold higher than those of the P‐deficient samples (0.8 μM P treatment), these extracts should be diluted differently to ensure an A_820_ reading of less than 1.0. A representative extract sample could be used to test whether the dilution is appropriate before performing a whole‐plate analysis.

### Optimal incubation time for Pi extraction

Unlike the conventional grinding method, the punched leaf disks were not ground because Pi was released following the immersion of the leaf disks in perchloric acid. To test for the optimal duration of Pi extraction, the leaves of cultivars accumulating high and low levels of Pi grown in 16 µM and 320 µM P were punched over their length with a hole puncher. The leaf disks were equally divided into five pools and stored at −80°C overnight. For the conventional Pi extraction, one pool of leaf disk samples was ground and extracted using the conventional grinding protocol, while the other four pools of samples were incubated in perchloric acid for 1, 2, 3, or 4 h, following the punching protocol. The Pi contents of the supernatants were then measured.

The results showed that 3 h of incubation time for the punching method yielded comparable results to the 30‐min incubation time used for ground tissue in the conventional method for both the 16 µM and 320 µM P treatments, and in both the high and low Pi‐accumulating cultivars (Appendix 3). In contrast, 1 h of incubation time yielded significantly lower Pi contents in the punching method samples, suggesting that 1 h is not sufficient to fully release Pi from the leaf disks. In addition, an extended incubation time (longer than 4 h) may result in the hydrolysis of organic P.

### Variability of Pi in different leaves under P‐sufficient and P‐deficient conditions

**Figure 1:**
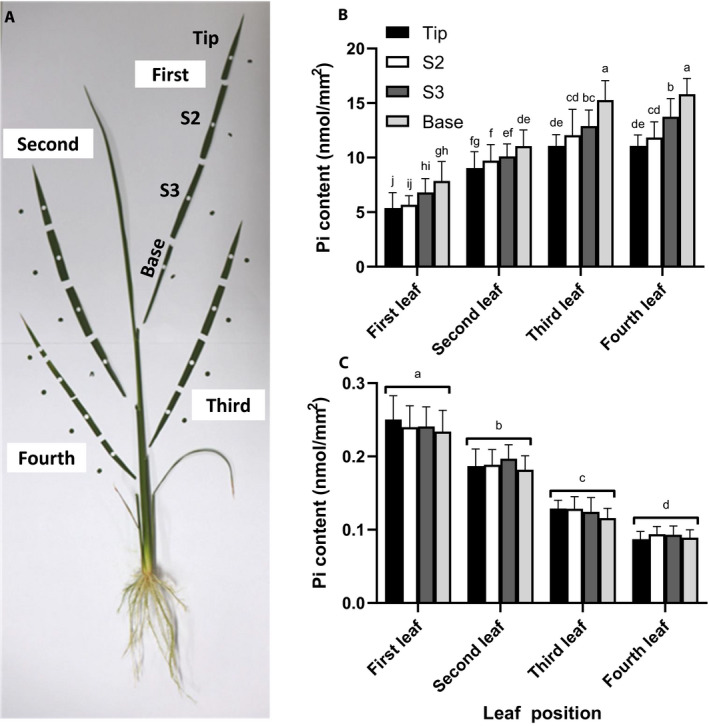
The effect of leaf age and position on the P content of the high Pi‐accumulating cultivar. (A) The fully expanded rice leaves were punched at different positions. (B, C) Pi contents of rice grown under P‐sufficient (B) and P‐deficient (C) conditions. Data are means ± SD (*n* = 12). Different letters indicate significant differences (*P* < 0.05) according to Duncan’s multiple range test. The experiments were repeated three times independently with similar results.

Our method requires only a small leaf sample for Pi measurement. As a 3‐mm hole puncher could cover the entire width of the long, narrow rice leaves at the seedling stages, we tested whether sampling at different positions along the leaf from the tip to the base affected the measured Pi contents (Fig. 1). We found that when the P supply was abundant (320 µM P), (1) more Pi accumulated in the leaf tips than the base, and (2) the Pi contents of older leaves (fourth fully expanded leaf) were greater than the Pi contents of younger leaves (Fig. 1B). In contrast, when the Pi supply was limited (16 µM P), our results showed that (1) the Pi content did not significantly vary along the leaf length, and (2) the Pi contents of younger leaves (first fully expanded leaf) were greater than the Pi contents in older leaves. This result is consistent with what has been previously reported regarding mobility of P and its translocation from older leaves to younger leaves (Rausch and Bucher, 2002) (Fig. 1C).

### Method validation

We compared the results of the two methods by extracting Pi from four leaf disks punched from the second section of the second fully expanded leaves (S2 of the second leaf on Fig. 1A) or from ground samples of the rest of the same leaves in order to (1) get enough tissue of the same leaf for the grinding method and (2) represent the averaged Pi contents from the whole leaf, which is typically performed when using the grinding method. Two rice cultivars grown in a wide range of P concentrations were included. The normalized Pi contents were calculated as nanomoles per leaf disk area (square millimeters) or micromoles per fresh weight (grams) for the punching method and the conventional grinding method, respectively. The average fresh weight of the leaf disks for each sample was also determined (Table 1) and used to convert the unit from nanomoles per leaf disk area to micromoles per fresh weight.

**Table 1:** Leaf disk weight (mg/disk) of the high and low Pi‐accumulating cultivars grown under different levels of P supply (320, 160, 80, 16, and 0.8 µM P). For each sample, the average fresh weight of a single leaf disk was calculated by weighing a pool of 80 leaf disks and dividing by the number of leaf disks. Data are means ± SD (*n* = 3 samples).

Cultivar	P supply (µM)	Fresh weight of leaf disk (mg)^a^
High Pi accumulation	0.8	0.887 ± 0.013 b
	16	1.025 ± 0.003 a
	80	1.037 ± 0.024 a
	160	1.036 ± 0.028 a
	320	1.039 ± 0.060 a
Low Pi accumulation	0.8	0.886 ± 0.009 b
	16	1.022 ± 0.022 a
	80	1.035 ± 0.051 a
	160	1.035 ± 0.025 a
	320	1.038 ± 0.045 a

^a^ Different letters indicate significant differences (*P* < 0.05) according to Duncan’s multiple range test.

**Figure 2:**
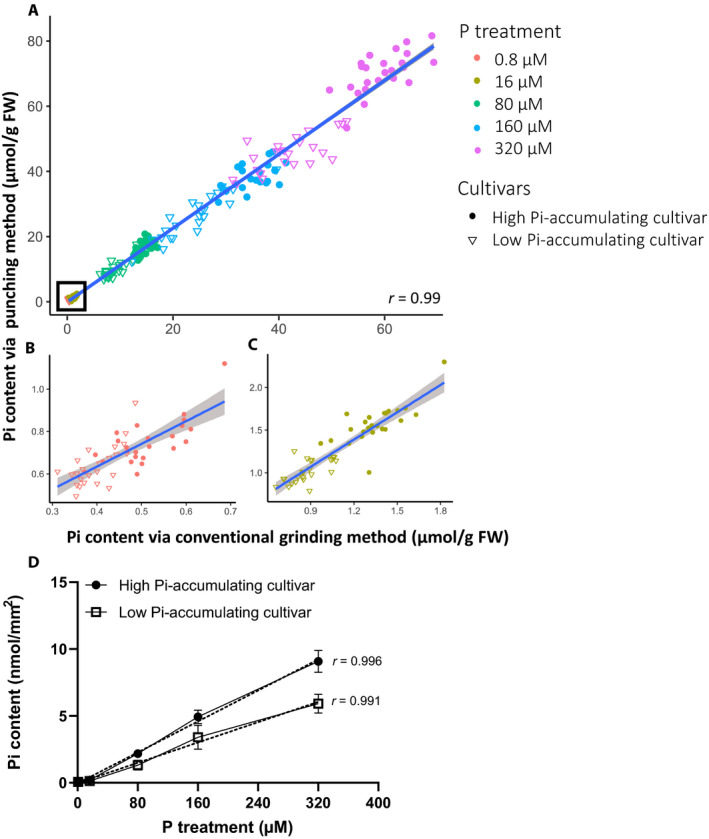
Pi contents of high and low Pi‐accumulating cultivars grown under different levels of P supply. (A–C) Linear regression plots of the Pi contents determined using the punching method and the conventional grinding method. The plants were grown under different levels of P supply (320, 160, 80, 16, and 0.8 µM P) (*n* = 24 per cultivar per P treatment). The data in the inset of (A) are shown in (B) for the 0.8 µM P treatment and in (C) for the 16 µM P treatment. FW, fresh weight. (D) Average Pi contents determined using the punching method, fitted with a linear regression. Data are means ± SD (*n* = 24). The correlation (*r*) was determined using Pearson’s correlation model. The experiments were repeated three times independently with similar results.

The measured Pi contents obtained using the punching method and the conventional grinding method were comparable (*r* = 0.99 when all P treatments were considered) (Fig. 2A). The *r* correlation values for the individual P treatments were 0.92, 0.92, 0.92, 0.90, and 0.78 for the 320, 160, 80, 16, and 0.8 µM P conditions, respectively (Fig. 2A–C). This result suggested that both methods yielded comparable and reproducible results in a wide range of P concentrations. However, the correlation declined when the Pi contents were low (under the 0.8 µM P treatment), as the plants showed visible symptoms of P deficiency including chlorosis in the lower leaves and stunted growth. This might be due to the Pi levels being too low to detect accurately in the analyzed samples. More leaf disks could be used to increase the amount of Pi extracted, and the number of leaf disks can be used for normalization by increasing the overall leaf area used to calculate Pi content. We tested linearity of the assay by varying the number of leaf disks, and a very high correlation (*r* = 0.99) was observed between the number of disks used and the Pi content detected (Appendix 4), illustrating the quantitative strength of the assay.

We also showed that the Pi contents in the second fully expanded leaves extracted using the punching method increased linearly with the P concentrations used in the treatment (*r* = 0.996 for the high Pi‐accumulating cultivar and 0.991 for the low Pi‐accumulating cultivar). The results showed that the leaves accumulate more Pi when the P supply was increased and that the high Pi‐accumulating cultivar had a higher Pi content than the low Pi‐accumulating cultivar at all P treatments tested (Fig. 2D).

We further compared the performance of our method with that of the inductively coupled plasma (ICP) technique. Samples with varying P contents were harvested by punching the first fully expanded leaves of rice seedlings (cv. Nipponbare) grown under varying P conditions. A pool of 300 leaf disks derived from 10 plants from the same treatment condition was divided into three technical replicates (each with four disks) for the determination of Pi using our protocol, and approximately 80 mg (fresh weight) was used for the total P determination using ICP–optical emission spectroscopy (ICP‐OES) (PQ9000 elite; Analytik Jena, Jena, Germany). A correlation analysis showed that the contents determined using each method were highly correlated (*r* = 0.97) (Appendix 5), although the values determined using ICP were greater than those determined using our protocol. This was because the ICP technique completely digested the samples in a microwave system and thus detected all P forms, including organically bound P, whereas the punching and grinding methods did not digest samples and the colorimetric assay detected only the soluble Pi forms (Kanno et al., 2016).

## CONCLUSIONS

The punching method presented here improved the throughput of the conventional Pi extraction and molybdate blue assay; one researcher can process thousands of samples in just 3 h of hands‐on time using 96‐well plates, multichannel pipettes, and microplate readers. Our tests showed that extracting the Pi contents without grinding, as well as normalizing the Pi contents with the leaf area, yielded comparable results to the conventional method that used ground samples and normalized the Pi contents to the biomass (Fig. 2A–C). Previous studies have also normalized the Pi content using the root length of *Arabidopsis thaliana* (L.) Heynh. seedlings grown vertically on agar plates on which the roots grow in one dimension (Ayadi et al., 2015; Kanno et al., 2016). This normalization technique may not accurately reflect the Pi contents in complex, three‐dimensional organs (e.g., flowers and large root systems), however, when the size or biomass of plant materials across the samples vary substantially.

Our results showed that Pi contents extracted from different positions on the leaves of a single plant could vary considerably (Fig. 1). This emphasized the need to select samples from the same position on the same leaf of each plant for the analysis, especially when the P supply is abundant. Comparing the Pi contents detected in the different positions in the plants grown in 320 µM P and 16 µM P (a 20‐fold decrease), the P contents varied by 21–33‐fold in the first leaf (youngest) and 125–177‐fold in the fourth leaf (older). When Pi is scarce, consideration should also be given to the sensitivity of Pi extraction and measurement using this method. Moreover, the leaf punching procedure could become more difficult as the P‐deficient leaves can be too small to handle with hole punchers. Thus, researchers should decide which leaf positions are most suitable in their specific experiments.

The difference between the Pi‐accumulation abilities between two rice cultivars grown under the same conditions suggested that this trait is likely determined by genetics. The cost of genomic studies keeps decreasing, making it more financially feasible to conduct phenomic studies to unravel the function of genes underlying agronomically important traits. Although this protocol had some limitations in plants with limited Pi content and was not optimized for other plant tissues, it could serve as a quick and simple method for the initial screening of large germplasm collections or to test a number of treatment variations before further validation with conventional methods.

## AUTHOR CONTRIBUTIONS

All authors conceived and designed the study. S.P. performed the experiments, prepared figures, and drafted the manuscript. J.C. wrote the final draft of the manuscript. All authors reviewed the manuscript and approved its final version.

## Appendix 1 Protocol for inorganic phosphate (Pi) extraction and measurement using the hole‐punching method with rice leaves.

### Parts list

96‐well plate

Dry ice

Ice

Glossy paper

Microplate reader

Multichannel pipette with tips

Hole puncher

Water bath

### Reagents

*Extraction buffer:*

- 5.5% (w/v) perchloric acid

*Molybdate blue reagents:*

- 0.4% (w/v) ammonium molybdate in 0.5 M H_2_SO_4_ (solution A)
- 10% ascorbic acid (solution B)

1. The molybdate blue reagent is freshly prepared by mixing both solutions (A : B = 6 : 1).
2. The solution is light sensitive.
3. Solution A should be stored at 4°C and newly prepared every four weeks.
4. Solution B should be freshly prepared and used within one day.

### Sample collection

1. Fold a rice leaf in half twice and place it over glossy paper.
2. Punch the leaf and glossy paper together with hole puncher.
3. Immediately transfer the leaf disks into a 96‐well plate, which is kept cold on dry ice.
4. Cover the 96‐well plate with aluminum foil and store at −80°C for the next step.

### Pi extraction

1. Add 200 µL of 5.5% (w/v) perchloric acid to each well containing leaf disks.
2. Cover the plate and incubate on ice for 3 h. Make sure all leaf disks are submerged in the solution.
3. Transfer the supernatant to a new 96‐well plate using a multichannel pipette. The supernatant can be diluted with 5.5% (w/v) perchloric acid by adding up to 80 µL of the final volume. Proceed to the next step for Pi quantification.

### Pi quantification

*Molybdate blue assay:*

1. Add 160 µL of molybdate blue reagent to 80 µL of the supernatant in each well using a multichannel pipette.
2. To generate the standard curve, prepare a serial dilution of known concentrations of KH_2_PO_4_ in the range of 0.1–40 µg/mL (equivalent to 0.74–294.12 µM). Add 160 µL of molybdate blue reagent to 80 µL of the phosphate standard.
3. Incubate the reactions at 40°C for 20 min.
4. Read the absorbance on the microplate reader at a wavelength of 820 nm and compare the values to the standard curve for quantification. The Pi content is calculated as the nanomoles per leaf disk area (square millimeters) or micromoles per gram fresh weight.
